# Validation of an obstetric fistula screening questionnaire: a case–control study with clinical examination

**DOI:** 10.1186/s12978-021-01317-2

**Published:** 2022-01-18

**Authors:** Chi Chiung Grace Chen, Annelise Long, Denis Rwabizi, Gerard Mbabazi, Ntwali Ndizeye, Blaise Dushimiyimana, Eugene Ngoga

**Affiliations:** 1 Department of Gynecology and Obstetrics, Johns Hopkins University School of Medicine, 4940 Eastern Ave, Baltimore, MD 21231 USA; 2 Department of Obstetrics and Gynecology, University of Rwanda, Kigali, Rwanda; 3 Rwanda Society of Obstetricians and Gynecologists, Kigali, Rwanda

**Keywords:** Vesicovaginal fistula, Rectovaginal fistula, Pelvic organ prolapse, Symptom-based screening questionnaire, Sensitivity, Specificity, Prevalence

## Abstract

**Background:**

Obstetric fistula (OF) is a significant cause of maternal morbidity in lower resource settings where women experience obstructed labor without timely access to skilled obstetric care. The true prevalence of OF is unknown; however, it is estimated to affect 2 to 3.5 million women globally. The Demographic and Health Surveys’ (DHS) Fistula Module includes the OF symptom questions most frequently used for prevalence estimates, but these questions have not been validated. The aim of this study is to validate a symptom-based screening questionnaire for OF, including a question in the DHS’ Fistula Module.

**Methods:**

With an international panel of fistula surgeons, we developed and face-validated a screening questionnaire that assessed for symptoms of lower urinary tract fistula (LUTF) and lower gastrointestinal tract fistula (LGTF), as well as urinary and fecal incontinence (UI, FI). We evaluated the discriminative ability of the questionnaire using a case–control study design in a 1:2:2 ratio: cases were parous women with fistula confirmed on examination, controls included parous women without fistula on examination, with and without UI symptoms. All women underwent screening for fistula symptoms and a physical examination, with examiners blinded to screening results.

**Results:**

Of the 367 Rwandan women who completed the questionnaires and underwent clinical examination, 59 women had LUTFs and 34 had LGTFs, 274 women were classified as controls with and without symptoms of UI. All LUTF screening questions performed well, including the DHS fistula question. The combination of two LUTF screening questions had the highest sensitivity (100%; 95% CI 94%, 100%), specificity (96%; 95% CI 93%, 98%), and area under the curve (AUC) (0.98). The combination of a LGTF screening question and FI question demonstrated the highest sensitivity (97%; 95% CI 85%, 100%), specificity (98%; 95% CI 95%, 99%) and AUC (0.98).

**Conclusions:**

Our OF screening questionnaire, including the DHS fistula question, demonstrated high sensitivities, specificities, and AUC.

**Supplementary Information:**

The online version contains supplementary material available at 10.1186/s12978-021-01317-2.

## Plain language summary

Obstetric fistula (OF) is a birth injury which may occur in women living in lower resource settings who experience obstructed labor (not progressing normally) without access to skilled obstetric care. This injury causes a woman to constantly leak urine and/or feces from her birth canal. As OF affects poor women who live far from healthcare it is difficult to fully understand how many women worldwide have an OF. Furthermore, although some Demographic and Health Surveys (DHS) include OF symptom questions, the accuracy of these questions in identifying women with OF has not been studied.

To more accurately determine which women may have an OF, we developed an OF screening questionnaire after consulting OF experts worldwide, which included a DHS OF question. We asked women from Rwanda this questionnaire and then examined these women to see if they have OF. Through this process we identified 59 women with a LUTF, 34 with a LGTF, and 274 without an OF. The best performing questions were able to identify women with LUTF 100% of the time and women with LGTF 97% of the time. We also showed that one DHS question detects women with LUTF and LGTF 100% and 85% of the time, respectively. Public health officials can now use the questions we studied to more accurately estimate how many women worldwide have OF, and best direct resources and skilled health care workers to the areas with the greatest need.

**Supplementary Information:**

The online version contains supplementary material available at 10.1186/s12978-021-01317-2.

## Background

Obstetric fistula (OF) is virtually eliminated in higher resource settings but remains a significant cause of maternal morbidity in lower resource settings, including Rwanda [1]. In these settings, OF results when women experience obstructed labor without timely access to skilled obstetric care [2]. Women with lower urinary tract fistulas (LUTF) and lower gastrointestinal tract fistulas (LGTF) have constant leakage of urine and feces and can also develop other medical issues including vaginal stenosis, genital ulcerations, foot drop from nerve injury, and amenorrhea [3]. Moreover, women with these conditions may be socially marginalized and suffer from psychiatric disorders.

The true prevalence of OF is unknown; however, it is widely cited to affect 2 to 3.5 million women globally, with an annual incidence of 50,000–100,000 using data primarily from hospital-based studies and expert opinions [4–6]. In Rwanda, the lifetime prevalence was estimated at 3.3% from the 2007 Demographic and Health Surveys (DHS) [7]. As in Rwanda, DHS in many countries have included OF symptom questions for prevalence estimations; however, the accuracy of these estimates are unknown as the questions have not been validated [8]. A 2015 systematic review of Southeast Asia and Africa reported OF incidences ranging from 0 to 4 cases per 1,000 deliveries while prevalences varied more widely from 0 to 81 cases per 1,000 women [9]. Most studies in the review utilized non-validated screening questionnaires. Women afflicted with OF may be both less able and less likely to seek care due to cultural restraints and fear of stigmatization, further calling into question the accuracy of these estimates [1, 4–6]. Without a validated screening questionnaire, accurate fistula diagnosis requires a clinical examination; however, a 2013 systematic review of OF prevalence found only ten out of thirteen studies utilized clinical confirmation [10].

Although clinical examination by skilled healthcare personnel is required to definitively diagnose OF, this is likely not the best way to study the prevalence and incidence of OF in a low resource setting. It is cost-prohibitive to send providers to examine entire communities as even the highest OF prevalence estimates suggest that OF is a low prevalence condition. A non-invasive, well-validated screening tool would likely increase the ability to effectively diagnose and treat women in underserved areas. Additionally, an accurate understanding of the distribution of OF prevalence could enable a more methodological allocation of health care resources to prevent and treat OF. In this study, we aimed to assess the validity of a symptom-based OF screening questionnaire in Rwanda. Although this screening questionnaire was previously piloted and preliminarily validated using a case–control design nested within a cross-sectional study in a low prevalence setting in rural Nepal, the low number of OF cases in this study population prevented any meaningful assessment of the predictive value of the questionnaire [11].

## Methods

### Fistula screening tool

The OF screening questionnaire, which included 15 test questions on LUTF and LGTF symptoms and 12 questions on other pelvic floor symptoms and basic reproductive history, was initially developed by the late Thomas Elkins (Professor, Johns Hopkins Department of Gynecology and Obstetrics) (Additional file 1: Appendix S1). The questionnaire also included urinary incontinence (UI) and fecal incontinence (FI) screening questions that have been validated in many populations [12, 13]. Prior to piloting the questionnaire in Nepal, face validation was performed and the questionnaire was modified accordingly with consultation from 8 expert fistula surgeons in the USA, Ethiopia, UK, and Nepal. To translate and culturally adapt the OF screening questionnaire for Rwanda, the questionnaire was first translated into two versions by Rwandan fistula surgeons and bilingual language experts. All translated versions were back-translated and then reviewed by local health workers and compiled to ensure that the final translated and culturally-adapted questionnaire reflected our original questionnaire and would be understandable to study participants. The translated questionnaire was pilot-tested on focus groups of women with clinically confirmed OF and women without OF, and further modified as appropriate.

We also included a question frequently used in DHSs in South Asia and Sub-Saharan Africa, including Rwanda, which addressed both LUTF and LGTF symptoms but had not been validated (Question 21, Table 2). The screening questionnaire also included questions for the interviewer on observations of wetness/ soiling and smells of urine/ feces on and around the study participants. Participants were verbally administered forms on demographics, medical and reproductive histories, as well as fistula histories and impact of fistula and UI symptoms on quality of life when appropriate.

Institutional review board approval was obtained from both Johns Hopkins Medicine (Baltimore, MD, USA) and the National Research Health Committee and the Rwanda National Ethics Committee (Kigali, Rwanda).

### Study design and sites

We designed and powered this case–control study to primarily evaluate the discriminative ability of questions on LUTF symptoms, as women with UI, which is far more prevalent than OF, may confuse these symptoms with LUTF symptoms. This is consistent with what has been reported in the literature and in our previous study in Nepal [11]. In that study, besides the 2 women with confirmed LUTFs, there were 65 false positive women who reported LUTF symptoms which were determined to be symptoms of UI. This mirrors our past clinical experiences as well as those of our international panel of content experts consulted on screening questionnaire development. Additionally, most large epidemiologic studies on OF prevalence or case series on OF have found less than 1% of all women with OF have an isolated LGTF (6). Our sample size calculation suggests that we need at least 58 LUTF cases to validate the screening questionnaire at 90% sensitivity and specificity with 10% margin of error at 0.05% error level at 90% power. We included two types of controls in a ratio of 1:2:2 (case:control:control): the first type of control was women who did not have a LUTF but did have symptoms of UI (Urinary Incontinence Controls, UC); the second type of control was women who did not have a LUTF and did not have symptoms of UI (Normal Control, NC).

**Fig. 1:**
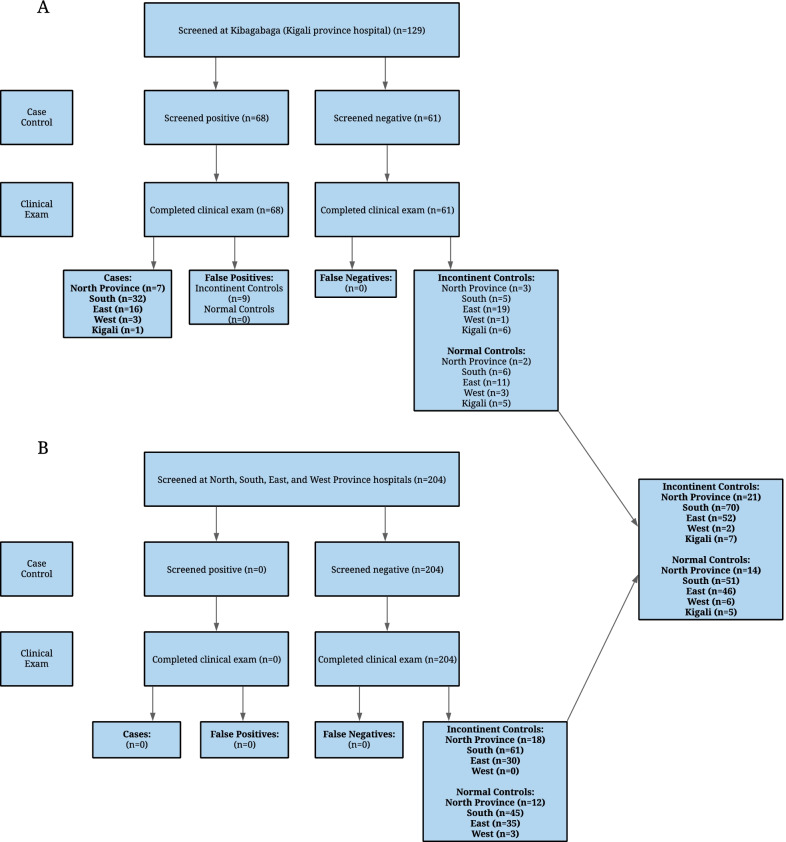
Study participation, screening results, and examination results

To a priori increase the likelihood of recruiting the needed number of LUTF cases, we conducted the case–control study at Kibagabaga district hospital, in Kigali, Rwanda; parous women with suspected OF symptoms are sent to this hospital by local health centres and health posts for evaluation and care triannually in a government sponsored program with support from a U.S. non-governmental agency (International Organization for Women and Development). After obtaining informed consent, interested women were screened for OF symptoms as well as UI and FI symptoms with our questionnaire (Fig. 1A). All study questionnaires, including the screening questionnaire, were verbally administered by preclinical medical students. Study participants then underwent clinical examinations by urogynecologists who were blinded to the screening questionnaire results. All women found to have an OF on clinical examination were classified as cases. Women found not to have an OF on examination were classified as UC or NC depending on if they had UI symptoms. Besides these women, to recruit the needed number of UC and NC, we also recruited parous women with and without UI symptoms at Kibagabaga and the other four provincial hospitals of Rwanda (North, West, East, South) in approximate proportion to the number of OF patients from each province (Fig. 1A, B). These women were screened for OF, UI, and FI symptoms with our questionnaire, and then examined by urogynecologists blinded to the questionnaire findings. All women received treatment according to their diagnoses.

### Clinical examination

All consenting participants underwent a clinical examination by board certified urogynecologists with experience in fistula care, who were blinded to the screening questionnaire results. To safeguard against missing OF cases, additional clinical tests were performed as needed, and confirmation of no OF was made by at least two experienced fistula surgeons. Radiologic studies such as intravenous pyelogram and/or barium enema studies were performed on a case-by-case basis. At the end of each day, we reviewed the screening questionnaire results and performed the aforementioned procedures, if not already performed, on any participants who screened positive for OF symptoms but were not found to have a clear fistula tract on initial examination.

### Analysis

Means (standard deviations), medians (ranges) were calculated as appropriate for continuous variables, and frequencies for categorical variables. Chi-square statistic for categorical variables and t-statistic for continuous variables were used to test for heterogeneity in basic baseline demographics and medical and reproductive histories for women with and without OF. The frequencies of the women’s self-reported OF symptoms from the screening questionnaire were compared to the clinical examination diagnosis. The sensitivity and specificity of the questions were calculated, along with their respective 95% confidence intervals [16]. The OF screening questions were compared to determine which questions, or combinations of questions, most accurately identified OF. Receiver Operating Characteristic (ROC) curves were plotted and the area under the curve (AUC) was also calculated for the questions [17, 18]. The data were analyzed in R version 3.3.1 [19].

To more accurately determine OF prevalence estimates, we applied the test characteristics we determined for the aforementioned DHS question to the lifetime prevalences of OF reported in DHS from 18 sub-Saharan countries, including Rwanda. We estimated the unobserved true prevalences utilizing:

$$P=\frac{(p + Sp - 1)}{(Se + Sp -1)}$$

P is the unobserved true prevalence, p the observed prevalence, Sp the question specificity, and Se the question sensitivity. This question had been asked among females aged 15–49 in 23 DHSs from 18 countries from 2005 to 2018; we used the data from the most recent surveys [14]. We excluded surveys that used a different sub-sample than females aged 15–49; however, as Rwanda is our country of interest, we did include the 2005 Rwanda DHS, which only asked OF questions to women who had given birth in the previous five years [15]. Our study is reported in accordance with Strengthening the Reporting of Observational Studies in Epidemiology (STROBE) recommendations. [20].

## Results

We administered the OF screening questionnaire to 367 parous women who consented to participate in the study at Kibagabaga district hospital (Kigali) and at the other four provincial hospitals across Rwanda (Fig. 1). The screening questionnaire took 30 min or less to administer. None of the women declined to answer any of the questions and 6.8% (4) women responded ‘don’t know’ to one or more of the questions. All 367 women underwent a clinical examination and we confirmed 59 LUTFs (36 bladder/ vagina, 20 bladder/ uterus, 3 ureteral/ vagina or uterus) and 34 LGTFs (rectum/ vagina); we reached our sample size of 58 LUTFs. No women had both a LUTF and a LGTF. The remaining 274 women were confirmed to not have a LUTF or LGTF. Of these women, 45% (122) had no symptoms of UI and were classified as NC and 55% (152) had symptoms of UI and were classified as UC; this allowed us to reach our 1:2:2 case:control:control ratio. Within our control patients, we did have three women from the NC group and three women from the UC group who also reported FI symptoms. Most LUTF and LGTF cases came from the Southern province (54% (32) and 35% (12), respectively).

Among all study participants, the mean age was 40.1 (sd 12.1) and the mean body mass index was 23.4 (sd 5.2) (Table 1). There was a median gravidity of 4 (range 1–14) and median rate of lifetime stillbirths of 0 (range 0–9). 29% of our study participants had received at least one cesarean section.

**Table 1:** Baseline characteristics of study participants by case–control status

	Normal controls (N = 122)	Urinary Incontinence controls (N = 152)	All controls (N = 274)	Lower urinary tract fistula cases (N = 59)	Lower gastro-intestinal tract cases (N = 34)	All fistula cases (N = 93)	Total (N = 367)	P-value**	P-value***
Age, mean(sd)	39.1 (10.9)	44 (13.1)	42 (12.4)	38 (11.3)	33.5 (7.9)	36.3 (10.3)	40.4 (12.1)	** < 0.001**	**0.03**
BMI (kg/m^2), mean(sd)	24 (6.5)	23.6 (4.7)	23.7 (5.6)	21.6 (2.9)	23.7 (3.8)	22.4 (3.4)	23.4 (5.2)	**0.0046**	** < 0.001**
Age at first pregnancy, mean(sd)	20.6 (3.7)	20.1 (3.7)	21.1 (3.8)	22 (4.1)	20.6 (3.1)	21.5 (3.8)	21.2 (3.8)	0.24	0.13
Lifetime pregnancies, median(range)	4 (1–4)	5 (1–13)	4 (1–14)	3 (1–12)	5 (1–8)	3 (1–12)	4 (1–14)	** < 0.001**	**0.003**
Lifetime vaginal deliveries, median(range)	4 (0–12)	5 (0–12)	4 (0–12)	1 (0–12)	3.5 (1–9)	2 (0–12)	3 (0–12)	** < 0.001**	** < 0.001**
Lifetime cesarean sections, median(range)	0 (0–6)	0 (0–5)	0 (0–6)	1 (0–4)	0 (0–1)	1 (0–4)	0 (0–6)	**0.002**	** < 0.001**
Lifetime stillbirths, median(range)	0 (0–2)	0 (0–7)	0 (0–7)	1 (0–9)	0 (0–2)	0 (0–9)	0 (0–9)	** < 0.001**	** < 0.001**
Lifetime miscarriage, median(range)	0 (0–6)	0 (0–4)	0 (0–6)	0 (0–8)	0 (0–2)	0 (0–8)	0 (0–8)	0.47	0.50
Lifetime abortion, median(range)	0 (0)	0 (0–2)	0 (0–2)	0 (0–1)	0 (0)	0 (0–1)	0 (0–2)	0.99	0.76
Previous Surgeries, N (%)									
Tubal Ligation	1 (0.8)	0 (0)	1 (0.4)	2 (3.4)	0 (0)	2 (2.15)	3 (0.8)	0.10	**0.03**
Cesarean section	28 (23.7)	30 (20.3)	58 (21.8)	44 (45)	3 (8.8)	47 (50)	105 (28.8)	** < 0.001**	** < 0.001**
Other pelvic surgery	7 (5.9)	9 (6.0)	16 (6.0)	10 (16.9)	2 (5.9)	12 (12.9)	28 (7.8)	**0.03**	**0.005**
Currently smoke, N (%)	2 (1.64)	7 (4.61)	9 (3.3)	6 (10)	2 (5.88)	8 (8.6)	17 (4.6)	**0.04**	**0.02**
Medical conditions, N (%)									
Diabetes	3 (2.5)	3 (2.0)	6 (2.21)	0 (0)	0 (0)	0 (0)	6 (1.6)	0.15	0.24
Hypertension	6 (5.1)	12 (8.1)	18 (6.7)	0 (0)	0 (0)	0 (0)	18 (5.5)	**0.001**	**0.04**
Gastrointestinal Issues	23 (19.5)	36 (24.3)	59 (22.2)	9 (15.25)	8 (23.5)	18 (19.4)	77 (21.1)	0.36	0.24
Other*	31 (26.3)	39 (26.6)	70 (26.3)	7 (11.9)	7 (20.6)	14 (15.1)	84 (23.0)	**0.02**	**0.02**

Bolded characters represent statistically significant values

*BMI* body mass index, *sd* standard deviation

*HIV/AIDs, Sinusitis, chronic pain, Asthma

**Comparing all fistula cases to all controls

***Comparing LUTF fistula cases to all controls

For the LUTF screening questions, the DHS fistula question (question 21) demonstrated the highest sensitivity, specificity, and area under the curve (Se = 1.00, 95% CI: 0.94–1.00; Sp = 0.95, 95% CI: 0.91–0.97; AUC = 0.97) (Table 2). When we evaluated different combinations of questions to assess for the most discriminating combination, we found the combinations of questions 5 and 21 (Se = 1.00, 95% CI: 0.94–1.00; Sp = 0.95, 95% CI: 0.91–0.97; AUC = 0.97), and questions 5 and 8a (Se = 1.00, 95% CI: 0.94–1.00; Sp = 0.96, 95% CI: 0.93–0.98; AUC = 0.98) performed the best. Questions 5 and 8a performed marginally better than the combination of questions 5 and 21. Importantly, there were no false negative cases. As expected, the UI symptom questions were less accurate in identifying women with LUTFs.

**Table 2:** Screening questionnaire test characteristics

Lower urinary tract fistula	Sensitivity (95% CI)	Specificity (95% CI)	AUC
5. When you are not urinating, do you routinely/consistently experience continuously dripping urine (through the birth canal/vagina) that you cannot stop/control?	0.95 (0.86, 0.99)	0.98 (0.96, 0.99)	0.97
6–3. Does the continuously dripping urine (through the birth canal/vagina) that you experience occur: Both all day and all night?	0.88 (0.77, 0.95)	0.99 (0.96, 1.00)	0.93
7–3. Do you leak urine all the time which wets your clothing: Both all day and all night?	0.83 (0.71, 0.92)	0.94 (0.91, 0.97)	0.89
8. Do you routinely/consistently experience sudden leakage of large amounts of urine?	0.83 (0.71, 0.92)	0.96 (0.93, 0.98)	0.90
8a. Does this urine leakage occur when you are not coughing or sneezing?	0.85 (0.73, 0.93)	0.97 (0.94, 0.98)	0.91
8b. Does this urine leakage occur without urgency?	0.80 (0.67, 0.89)	0.97 (0.94, 0.98)	0.88
21. Sometimes a woman can have a problem such that she experiences a constant leakage of urine or feces from her birth canal/vagina during the day and night. This problem usually occurs after a difficult childbirth, but may also occur after a sexual assault or after a pelvic surgery Have you ever experienced (now or in the past) a constant leakage of urine and/or stool from your birth canal/vagina during the day and night?*	1.00 (0.94, 1.00)	0.95 (0.91, 0.97)	0.97
5 and 7–3	0.97 (0.88, 1.00)	0.94 (0.90, 0.96)	0.95
5 and 8a	1.00 (0.94, 1.00)	0.96 (0.93, 0.98)	0.98
5 and 21	1.00 (0.94, 1.00)	0.95 (0.91, 0.97)	0.97
6–3 and 21	1.00 (0.94, 1.00)	0.94 (0.70, 1.00)	0.96
7–3 and 21	1.00 (0.94, 1.00)	0.96 (0.80, 1.00)	0.94
8 and 21	1.00 (0.94, 1.00)	0.96 (0.78, 1.00)	0.94
8a and 21	1.00 (0.94, 1.00)	0.95 (0.76, 1.00)	0.95
8b and 21	1.00 (0.94, 1.00)	0.95 (0.76, 1.00)	0.94
16. When not being treated for infection (e.g. urinary tract infection), in a typical month, do you ever lose urine during sudden physical exertion, lifting, coughing or sneezing?	0.76 (0.63, 0.86)	0.66 (0.60, 0.71)	0.71
17. When not being treated for infections, in a typical month, do you ever experience such a strong and sudden urge to urinate that you leak before reaching the toilet?	0.59 (0.46, 0.72)	0.53 (0.46, 0.59)	0.56
16 and 17	0.88 (0.77, 0.95)	0.45 (0.39, 0.51)	0.67
International Consultation on Incontinence- Urinary Incontinence When does urine leak?			
Leaks before you can get to the toilet	0.63 (0.49, 0.75)	0.61 (0.55, 0.67)	0.57
Leaks when you cough or sneeze	0.64 (0.51, 0.76)	0.73 (0.67, 0.78)	0.62
Leaks when you are asleep	0.80 (0.67, 0.89)	0.93 (0.89, 0.95)	0.83
Leaks when you are physically active/exercising	0.71 (0.58, 0.82)	0.88 (0.84, 0.92)	0.75
Leaks when you have finished urinating and are dressed	0.39 (0.27, 0.53)	0.93 (0.89, 0.96)	0.71
Leaks for no obvious reason	0.86 (0.75, 0.94)	0.94 (0.90, 0.96)	0.86
Leaks all the time	0.78 (0.65, 0.88)	0.96 (0.93, 0.98)	0.88

Lower gastrointestinal tract fistula	Sensitivity (95% CI)	Specificity (95% CI)	AUC
9. When you are not having a bowel movement, do you routinely/consistently experience feces passing through the birth canal/vagina that you cannot stop/control?	0.88 (0.73, 0.97)	1.00 (0.99, 1.00)	0.94
18. We would like to ask you about any leakage of feces. Please do not include problems during short-term illness (such as a flu or virus/ diarrhea). Do you have problems with leakage of feces from the anus (accidents or soiling because of the inability to control the passage of feces until you reached a toilet)?	0.65 (0.46, 0.80)	0.98 (0.95, 0.99)	0.81
21. Sometimes a woman can have a problem such that she experiences a constant leakage of urine or feces from her birth canal/vagina during the day and night. This problem usually occurs after a difficult childbirth, but may also occur after a sexual assault or after a pelvic surgery Have you ever experienced (now or in the past) a constant leakage of urine and/or stool from your birth canal/vagina during the day and night?*	0.85 (0.68, 0.95)	0.94 (0.70, 1.00)	0.90
9 and 18	0.97 (0.85, 1.00)	0.98 (0.95, 0.99)	0.98
9 and 21	0.91 (0.76, 0.98)	0.95 (0.91, 0.97)	0.95
18 and 21	0.91 (0.76, 0.98)	0.92 (0.89, 0.95)	0.94

*AUC* area under the curve

*Demographic health survey fistula screening question

While both LGTF screening questions (question 9 and question 21) performed well, question 9 had the highest sensitivity and specificity (Se = 0.88, 95% CI: 0.73–0.97; Sp = 1.00, 95% CI: 0.99–1.00; AUC = 0.94) (Table 2). However, both questions still missed at least three LGTF cases (three false negatives). Interestingly, although question 18 on FI symptoms performed poorly alone (Se = 0.65, 95% CI: 0.46–0.80; Sp = 0.98, 95% CI: 0.95–0.99; AUC = 0.81), when added to question 9, only one LGTF case screened false negative, resulting in higher sensitivity and AUC than both questions alone (Se = 0.97, 95% CI: 0.85–1.00; Sp = 0.98, 95% CI: 0.95–0.99; AUC = 0.98). The one false negative case was a woman who had a previous LGTF repair but whose fistula either recurred or persisted. However, at the time of clinical examination she denied bothersome symptoms and declined reoperation on her fistula.

Applying the DHS fistula question’s (question 21) test characteristics to the observed prevalences reported by the DHS did not result in meaningful estimates as the reported prevalence estimates of 1–40 cases per 1,000 were far lower than our false positive rate (50 cases per 1000) (Table 3). A screening question would need a specificity of 99.9% in order to interpret the true prevalence of the lowest observed prevalence of 1 case per 1,000 women in Burkina Faso and Senegal.

**Table 3:** True prevalence estimates using demographic and health surveys

DHS Survey Country & Year	Sample size	Observed prevalence (per 1000 women)	True unobserved prevalence (per 1000 women)
Benin 2011–12	16,599	7	− 45
Burkina Faso 2010	17,062	1	− 52
Burundi 2016–17	17,269	8	− 44
Cameroon 2011	15,419	4	− 48
Congo 2011–12	10,818	3	− 49
Ethiopia 2016	15,683	4	− 48
Guinea 2018	10,874	40	− 9
Kenya 2014	14,737	10	− 42
Malawi 2015	24,562	6	− 46
Mali 2018	10,519	4	− 48
Nigeria 2008	33,317	4	− 48
Rwanda 2005	5420	30	− 18
Senegal 2010–11	15,688	1	− 52
Sierra Leone 2013	16,543	7	− 45
Tanzania 2010	10,136	6	− 46
Togo 2013	9,474	10	− 42
Uganda 2016	18,506	14	− 38
Yemen 2013	16,457	8	− 44
Zambia 2018	13,683	2	− 51
Country A		50	0
Country B		60	11
Country C		70	21

## Discussion

### Main findings

We developed and validated a symptom-based OF screening questionnaire that was highly discriminative for LUTF and LGTF symptoms. Importantly, we were able to determine the test characteristics of a previously non-validated OF screening question used by the DHS to estimate fistula prevalence. We further created a pared-down version of the OF screening questionnaire with the top performing combination of questions: two LUTF symptom questions, one LGTF question, and one FI question (Additional file 2: Appendix S2). As the entire screening questionnaire (27 questions) used during the study was able to be verbally administered in 30 min or less, it is likely the revised questionnaire (8 questions) can be administered in 10 min or less.

We have now demonstrated the feasibility and validity of our screening questionnaire in two settings, Rwanda and Nepal. In Nepal, we studied the questionnaire using a case–control study nested within a cross-sectional study design involving almost 17,000 women. In Nepal, the screening questionnaire demonstrated high sensitivities 100% (95% CI 34.2%, 100.0%) and specificities 86.9% (95% CI 83.3%, 89.9%), for LUTF symptoms and high sensitivities 100% (95% CI 20.7%, 100%) and specificities 99.8% (95% CI 98.6%, 100%), for LGTF symptoms. However, the critical limitation of validating the OF screening questionnaire in this setting was a low OF prevalence estimate (12 per 100,000 parous, reproductive-age women (95% CI 3, 43)). Therefore, our statistics were driven by the large number of women without OF symptoms. Although we cannot estimate prevalence using the case–control design of our study in Rwanda, the screening questionnaire appears to also perform well in this setting with a higher prevalence of OF. There were no false negative LUTF cases in either setting and only one false negative LGTF in Rwanda. The false negative LGTF in Rwanda was a woman with a recurrent fistula but did not report symptoms and ultimately did not elect to undergo treatment. The number of false positives was also relatively low in both settings (53 LUTF in Nepal, 9 LUTF in Rwanda, no LGTF in either setting), which may be equally pertinent to the utility of this screening questionnaire. These false positives can be attributed to the low prevalence nature of OF and the high prevalence nature of UI (pooled prevalence of 30% in LMIC), and the overlapping symptoms in the two conditions [21]. In Nepal, 77% (41) of the false positives had UI, with 83% of these women experiencing severe or very severe UI based on the Sandvik Incontinence Severity Index [22]. Of those women without UI, 23% (11) were diagnosed with vaginal prolapse and 49% (26) had abnormal vaginal discharge. Only 6% (3) of false positives were without UI, vaginal prolapse, or vaginal discharge. Similar to these findings, 100% of the false positives in Rwanda had some type of UI: 6 with stress urinary incontinence (67%), and 3 with both stress and urge urinary incontinence (33%); all 9 reported severe to very severe UI as measured by the Sandvik Incontinence Severity Index [22]. Five of the false positive women (56%) underwent previous OF repairs.

### Interpretation

Our OF screening questionnaire can be used to identify cases of OF that require medical attention and to empower local providers with a dependable tool and further understanding of OF symptoms. As community outreach programs such as radio campaigns have been shown to be an effective tool to increase OF treatment seeking [23, 24], these campaigns could offer a hotline for women who suspect they have OF. Hotline operators could administer our validated screening questionnaire, and refer women who screened positive to the nearest center for fistula evaluation [25]. This type of screening approach was utilized in Nigeria and Uganda, using different questions (not published), through an EngenderHealth managed USAID Fistula Care Plus Project; this strategy was useful in addressing barriers to OF diagnosis and treatment [26].

There are limited studies addressing the validation of screening tests for OF. The OF Community Based Assessment Tool aimed to screen women for OF symptoms in a community setting and involved two parts: a non-scripted client interview by a community health worker (CHW) followed by scripted questions on LUTF and/or LGTF symptoms, potential causes of OF symptoms, and timing of both cause and symptoms [27]. The CHWs decided which scripted questions to ask based on their assessment of patient symptoms after the initial non-scripted interview. Therefore, the performance of this tool relies on the judgment of CHWs. Although the authors reported testing this tool in a community-based setting in Kenya, they did not publish findings on test characteristics. In our study, all women were asked the same screening questions. We did use preclinical medical students, but they were instructed to ask the questions verbatim. Furthermore, in Nepal, our screening questionnaire was administered by our team of female field interviewers with at most a secondary-school education.

### Strengths and limitations

The strengths of this study include face validation of the OF screening questionnaire with an international team of fistula surgeons as well as the use of rigorous methodology to translate/ back translate and culturally adapt the OF screening questions for Rwanda. We used a case–control study design to identify an adequate number of cases to evaluate the test characteristics of various OF screening questions on a group of women with a diverse range of specific OF diagnoses, symptoms, and medical histories. However, this study may be subject to spectrum bias as we were only able to evaluate this questionnaire in a hospital-based setting and it may be that only women with the most severe symptoms presented for evaluation. However, the questionnaire was designed to capture women with OF symptoms bothersome enough to warrant care seeking, leading to potential utilization of healthcare resources. Therefore, it may not be consequential from a public health perspective if the questionnaire does not capture women who have less bothersome OF symptoms. Furthermore, these screening questions were previously studied on a population level in rural Nepal and demonstrated similar test characteristics.

Although we were able to determine the test characteristics of a previously non-validated OF screening question used by the DHS to estimate fistula prevalence; there are limitations to applying these test characteristics to past and future DHS prevalence estimates. Our study population was nonrandom, consisting of women either presenting with suspected OF, or at a district hospital for an unrelated condition or family support. In contrast, the DHS is a household survey that utilizes random cluster sampling. The screening question may be interpreted differently in a health center versus household setting. Women answering this question on a DHS may be subject to social desirability bias, as the survey administrators are not health care professionals. These limitations notwithstanding, this is the first DHS Fistula Module question that has been clinically validated; we have determined that the question the DHS utilizes to estimate lifetime prevalence is accurate.

## Conclusions

This OF screening questionnaire has been studied in two countries using two different study designs with very different frequencies of OF and has been shown to be feasible to administer and highly discriminative for OF symptoms. Public health officials can utilize this questionnaire to more accurately report the global disease burden from OFs and determine health care resource needs.

## Supplementary Information

**Additional file 1: Appendix S1.** Obstetric Fistula Screening Questionnaire

**Additional file 2: Appendix S2.** Updated Obstetric Fistula Screening Questionnaire.

## Data Availability

The datasets used and/or analyzed during the current study are available from the corresponding author on reasonable request.

## Abbreviations

OF: Obstetric fistula
LUTF: Lower urinary tract fistulas
LGTF: Lower gastrointestinal tract fistulas
DHS: Demographic and Health Surveys
UI: Urinary incontinence
FI: Fecal incontinence
AUC: Area under the curve
ROC: Receiver operating characteristic
UC: Urinary incontinence controls
NC: Normal controls
STROBE: Strengthening the Reporting of Observational Studies in Epidemiology
