# Supplementary material for: Validation of an obstetric fistula screening questionnaire: a case–control study with clinical examination
Source: Reprod Health. 2022 Jan 18;19:12. doi: 10.1186/s12978-021-01317-2 (PMC8764794; doi:10.1186/s12978-021-01317-2)

Appendix S1: Obstetric Fistula Screening Questionnaire

**Rwanda Fistula Screening Form**

**Interviewer ID**

**DD MM YY**

**Date**

**Province (Intara) District (Akarere) Sector (Umurenge)**

**________________________ ________________________ ________________________**

**Cell (Akagari) Village (Umudugudu)**

**________________________ ________________________**

**IOWDNUM**

**First names Last name**

_________________________ _________________________

**Recruitment Site**

**1=Yes
6=Refused (STOP)**

**Consent Status**

______________________________________

**For all questions: 6 or 66=Refused, 9 or 99=Don’t Know or Cannot Determine**

| **No.** | **Question** | **Coding** | **Response** |
| --- | --- | --- | --- |
| **1.** | What is your age? | Number of years |  |
| **2.** | How many times have you been pregnant?  (Response here should equal c+d+e+f) | Number of times  If “00” (Go to Q5) |  |
|  | **a.** How many vaginal deliveries? | Number |  |
|  | **b.** How many C-sections? | Number |  |
|  | **c.** How many live births? | Number |  |
|  | **d.** How many dead babies (stillbirths) have you delivered? | Number |  |
|  | **e.** How many miscarriages? | Number |  |
|  | **f.** How many terminations/abortions? | Number |  |
| **3.** | Are you now pregnant? | 0=No (Go to Q4) 1=Yes |  |
|  | **a.** If yes, how many weeks pregnant are you now?  (ASK HOW MANY WEEKS AGO WAS HER LAST MENSTRUAL PERIOD TO GET NUMBER OF WEEKS PREGNANT) | Number of weeks |  |
| **4.** | How long ago was your last delivery? | Number of years  AND  Number of months |  |
|  | **a.** Was it a vaginal delivery? | 0=No 1=Yes |  |
|  | **b.** Was it a C-section? | 0=No 1=Yes |  |
|  | **c.** Was the baby dead (stillborn)? | 0=No 1=Yes |  |
| **5.** | When you are not urinating, do you routinely/consistently experience continuously dripping urine (through the birth canal/vagina) that you cannot stop/control? | 0=No 1=Yes |  |
| **6.** | Does the continuously dripping urine (through the birth canal/vagina) that you experience occur: | 0=Does not occur  1=All day  2=All night  3=Both |  |
| **7.** | Do you leak urine all the time which wets your clothing? | 0=No 1=Yes, all day  2=Yes, all night  3=Both |  |
| **8.** | Do you routinely/consistently experience sudden leakage of large amounts of urine that wets your clothing not with urgency nor during sudden physical exertion, lifting, coughing or sneezing? | 0=No  1=Yes |  |
| **9.** | When you are not having a bowel movement, do you routinely/consistently experience feces passing through the birth canal/vagina that you cannot stop/control? | 0=No  1=Yes |  |
| **10.** | (Do not ask Question 10 if the woman’s response to questions 5-9 WERE ALL “0=No”)  Did any of the urine and/or feces leakage issues described above occur:  (RECORD all that apply)  (GO TO Q12, UNESS IT OCCURRED AFTER A DELIVERY, THEN GO TO Q11) | 0=No leakage (Go to Q12)  1=After no preceding event (Go to Q12)  2=After delivery (Go to Q11)  3=After rape/sexual assault (Go to Q12)  4=After pelvic surgery (Go to Q12)  5=After some other event (specify): | A  B  C  D |
| **11.** | (Do not ask Question 11 if the woman’s response to questions 5-9 WERE ALL “0=No”)  Was this baby born alive? | 0=No 1=Yes |  |
|  | **a.** Was it a vaginal delivery? | 0=No 1=Yes |  |
|  | **b.** Was it a C-section? | 0=No 1=Yes |  |
|  | **c**. Was it a normal labor and delivery, or a very difficult labor and delivery? | 0=Normal  1=Very difficult |  |
| **12.** | Have your heard of, “fistula” before? | 0=No (Go to 16) 1=Yes (Go to 13) |  |
| **13.** | Do you think you have a fistula/ obstetric fistula? | 0=No 1=Yes |  |
|  | **a.** Is it a bladder fistula? | 0=No 1=Yes |  |
|  | **b.** Is it an anal fistula? | 0=No 1=Yes |  |
| **14.** | Have you been told you have a fistula/ obstetric fistula by a health worker/doctor? | 0=No 1=Yes |  |
|  | **a.** Were you told it was a bladder fistula? | 0=No 1=Yes |  |
|  | **b.** Were you told it was an anal fistula? | 0=No 1=Yes |  |
| **15.** | How many fistula repairs have you had? (If 0, Go to Q15) | Number of times |  |
|  | **a.** How many bladder fistula repairs? | Number of times |  |
|  | **b.** How many anal fistula repairs? | Number of times |  |
| **16.** | When not being treated for infection (e.g. urinary tract infection), in a typical month, do you ever lose urine during sudden physical exertion, lifting, coughing or sneezing? | 0=No 1=Yes |  |
| **17.** | When not being treated for infections, in a typical month, do you ever experience such a strong and sudden urge to urinate that you leak before reaching the toilet? | 0=No 1=Yes |  |
| **18.** | We would like to ask you about any leakage of feces.  Please do not include problems during short-term illness (such as a flu or virus/ diarrhea). Do you have problems with leakage of feces from the anus (accidents or soiling because of the inability to control the passage of feces until you reached a toilet)? | 0=No  1=Yes |  |
| **19.** | Do you usually have a bulge/mass or something bulging out that you can see or feel in your birth canal/ vaginal area? | 0=No 1=Yes |  |
| **20.** | Have you had any previous surgeries in the lower abdomen, birth canal/vagina, bladder or anus such as:  (RECORD ALL THAT APPLY) | 0=None  1= Fistula repair  2=Cesarean section  3=Contraceptive surgery/ sterilization  4=Hysterectomy  5=Prolapse surgery  6=Abortion  7=Other, specify:  ______________________ | A  B  C  D  E  F  G |
| **21.** | Sometimes a woman can have a problem such that she experiences a constant leakage of urine or feces from her birth canal/vagina during the day and night. This problem usually occurs after a difficult childbirth, but may also occur after a sexual assault or after a pelvic surgery.  Have you ever experienced (now or in the past) a constant leakage of urine and/or stool from your birth canal/vagina during the day and night? | 0=No  1=Urine  2=Feces  3=Both |  |
| **22.** | ICIQ Score |  |  |
|  | **a.** How often do you leak urine? | 0=Never  1=About once a week or less often  2=Two or three times a week  3=About once a day  4=Several times a day  5=All the time |  |
|  | **b.** We would like to know how much urine you think leaks. How much urine do you usually leak (whether you wear protection or not)?  IF QUESTION IS REFUSED PLEASE USE ‘66’ CODE NOT ‘6’ CODE | 0=None  2=A small amount  4=A moderate amount  6=A large amount |  |
|  | **c.** Overall, how much does leaking urine interfere with your everyday life?  WRITE NUMBER BETWEEN 0 (NOT AT ALL) AND 10 (A GREAT DEAL) |  |  |
|  | 0 1 2 3 4 5 6 7 8 9 10  Not at all A great deal | |  |
|  | (SUM SCORES FROM **22a, b, c** ABOVE) | Sum |  |
| **23.** | When does urine leak?  (SELECT ALL THAT APPLY) | 0=Never, urine does not leak  1=Leaks before you can get to the toilet  2=Leaks when you cough or sneeze  3=Leaks when you are asleep  4=Leaks when you are physically active/exercising  5=Leaks when you have finished urinating and are dressed  6=Leaks for no obvious reason  7=Leaks all the time | A  B  C  D  E  F  G |
| **24.** | Please indicate the severity of your urine leakage on the picture.  (SHOW PICTURE) | 1=Picture 1  2=Picture 2  3=Picture 3 |  |

**INTERVIEWER OBSERVATION**

| **No.** | **Question** | **Coding** | **Response** |
| --- | --- | --- | --- |
| **25.** | Do you think you smell the subject’s own urine and/or feces around her? | 0=No  1=Urine  2=Feces  3=Both |  |
| **26.** | Do you think the subject’s clothing is wet with her own urine and/or feces? | 0=No  1=Urine  2=Feces  3=Both |  |
| **27.** | Do you think you see wetness around the subject from her own urine and/or feces? | 0=No  1=Urine  2=Feces  3=Both |  |


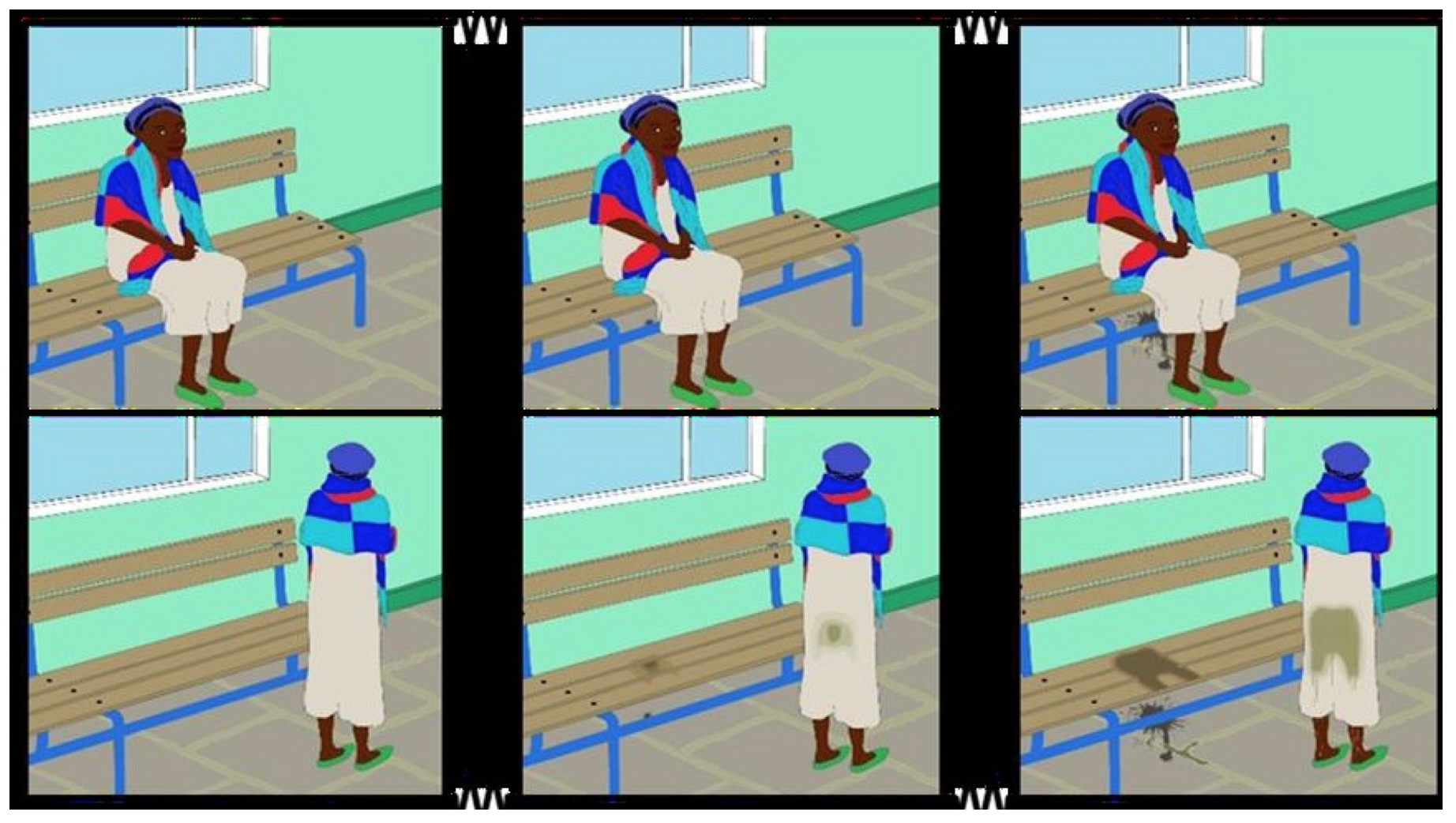

Supplement: Supplementary file 1 — Additional file 1: Appendix S1. Obstetric Fistula Screening Questionnaire [file 12978_2021_1317_MOESM1_ESM.docx]
