# Supplementary material for: Validation of an obstetric fistula screening questionnaire: a case–control study with clinical examination
Source: Reprod Health. 2022 Jan 18;19:12. doi: 10.1186/s12978-021-01317-2 (PMC8764794; doi:10.1186/s12978-021-01317-2)
Supplement: Supplementary file 2 — Additional file 2: Appendix S2. Updated Obstetric Fistula Screening Questionnaire. [file 12978_2021_1317_MOESM2_ESM.docx]

Appendix 2: Updated Obstetric Fistula Screening Questionnaire
